# Protocol for Electrical Conductivity Signal Collection and Processing in Scoliosis Surgery

**DOI:** 10.1155/2023/9955520

**Published:** 2023-11-07

**Authors:** Elie Saghbiny, Jimmy Da Silva, Celia Chaimi, Thibault Chandanson, Raphael Vialle

**Affiliations:** ^1^Hôpital Armand-Trousseau, APHP, Paris, France; ^2^ISIR-Institut de systèmes intelligents et de robotique, Sorbonne University, Paris, France; ^3^SpineGuard, Vincennes, France

## Abstract

**Introduction:**

Pedicle screw placement is a common procedure in spinal surgery. The misplacement rate with lateral and medial cortical perforation is 5–11%. Several techniques are used to decrease this rate. Many studies proved that electrical conductivity increases accuracy during pedicle screw placement but no study has interpreted conductivity values.

**Methods:**

The data are collected from patients operated for scoliosis in a single university hospital. After the posterior surgical approach is made, each pedicle is prepared classically. Instead of the classic curved pedicle probe, the surgeon uses a probe with the same shape that measures the conductivity at its tip. Conductivity values are recorded through a Bluetooth application. Each pedicle trajectory is then qualified after manual palpation with a feeler. A trajectory is qualified as optimal when palpation shows a bone tunnel without any breach, breached when there was a breach, and a modification of the probe direction was needed. A trajectory that does not meet the abovementioned definitions is excluded from the statistical analysis.

**Results:**

21 patients with 457 pedicles are recorded. The average age of the population is 14.71 ± 1.86 years. 17 patients (81%) have idiopathic adolescent scoliosis. One patient has Rett syndrome, one has hypotonia, one has cerebral palsy, and one has congenital malformation. The depth of the instrument is measured semiautomatically. This technique is validated when compared with the manual technique using the Bland–Altman agreement method (mean differences = −0.279 mm, upper limit = 2.2 mm, and lower limit = −2.7 mm) and Deming regression (slope = 1.06 ± 0.004).

**Conclusion:**

This study establishes a protocol to collect electrical conductivity signals in spine surgery with synchronization to the depth of the instrument. Real-time conductivity signal feedback alerts the surgeon of a probable breach in the spinal canal, so he can change the direction of the pedicle aim.

## 1. Introduction

Spine surgery is becoming one of the most important fields of research. Posterior spine fusion is the current gold standard for the treatment of spinal pathologies where fusion is needed. Correction of deformity has evolved since the Harrington rod system; first, sublaminar wires were used, then multiple hooks, and now pedicle screws construct. Screws act as a powerful anchor with the ability to achieve better correction of the spine [1]. Freehand pedicle preparation is a challenging procedure, especially in spine deformities. The failure rate during pedicle screw placement depends on the surgeon's experience and the patient's morphology. In a systematic review, the overall reported rate of mispositioning is around 11% [1]. The rate of mispositioning depends on the evaluating technique. When evaluated with postoperative plain radiographs, 4.2% of screws were mispositioned [2–4]. However, in studies in which postoperative computed tomography is performed, the mispositioning rate is 15.7% [5–7]. It should be noted that the definition of a mispositioned screw varies according to the studies. For some studies, any breach is a misposition [5, 6]; for others, it requires a breach of more than 2 mm [2].

The placement of pedicle screws may be more difficult in patients with significant spinal deformities because of the thinness and deformity of the pedicles [8]. The malpositioning of the screws can lead to numerous complications such as neurological complications in the event of a dural breach by direct trauma to the spinal cord with the screw or in the event of an epidural hematoma [5, 6, 9, 10]. Pulmonary and vascular complications can also occur in case of anterior and lateral breaches [6, 7].

Given the high rate of complications, monitoring of pedicle screw placement is mandatory during the operation. Many techniques are used to assist the surgeon during pedicle preparation. These techniques include preoperative planning, fluoroscopy during pedicle preparation, navigation use, and robotic assistance [11, 12]. All of these techniques are based on either ionized radiation to the patient or geometric registration of the patient's anatomical landmarks intraoperatively. In this case, any change in the patient's position during the operation may result in misplacement of the screws. Neuromonitoring with sensory and motor evoked potentials gives feedback about the spinal cord without any information about the screw placement [13].

Electrical conductivity of biological structures is a functional technique used in spine surgery that has the advantage of providing live feedback during surgery [14, 15]. This is based on the principle that osseous tissue can be considered an inhomogeneous and highly anisotropic material containing less conductive bone minerals and more conductive soft tissue such as blood vessels and other bodily fluids. The bone conductivity is thus mostly linked to the amount of blood in the bone, which evolves relatively to its density/porosity. As a result, cortical bone is less conductive than spongious bone. Pedicle probes embedding a bipolar conductivity sensor at their tip have been developed to provide local electrical conductivity measurements for spine surgery [16]. The embedded sensor measures the electrical conductivity in the surrounding tissues thanks to two separate electrodes, allowing relative differentiation of tissue conductivity. The conductivity measurement is delivered to the surgeon via audio feedback. The cadence and pitch of the audio signal increase when getting closer to higher conductivity tissues such as cerebral-spinal fluids and soft tissues [14, 15, 17]. Recently, the signal is transmitted wirelessly and displayed through an application.

**Figure 1:**
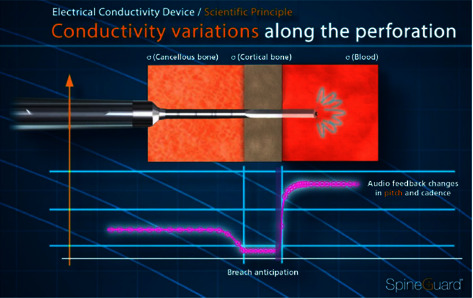
Expected signal variation concerning the different anatomical parts.

Figure 1 shows the expected signal variation concerning the different anatomical parts encountered during a breaching pedicle preparation. When reaching the cortical outer bone layer, the conductivity drops. Then, if the outer layer is broken, and the tip approaches the outside of the bone, the conductivity will increase drastically because of the proximity to conductive tissues (blood, muscles, and veins).

To date, no study has interpreted the conductivity values of the electrical signal. This study aims to develop a protocol to collect and analyze conductivity in patients operated on for scoliosis.

## 2. Materials and Methods

### 2.1. Protocol and Setup to Record Surgeries

The patients included in the study were first-time scoliosis patients. All patients were operated on at the same university center by the same surgeon. All legal representatives of patients consented to data recording and manipulation. Twenty-one patients (18 males and 3 females) were included in the study. Four patients had hypotony, Rett syndrome, cerebral palsy, and congenital malformation. Demographic characteristics of the population are summarized in Table 1.

**Figure 2:**
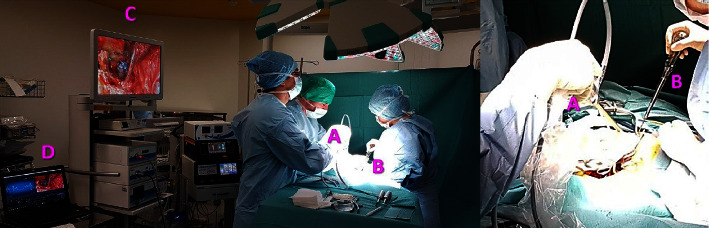
Setup used in the operating room: (A) endoscope, (B) PediGuard, (C) arthroscopy column, and (D) laptop recording synchronous video and electrical conductivity data.

The posterior aspect of the spine was exposed by a posterior approach. Each pedicle was prepared classically. Entry point preparation was performed with a 9 mm bone awl. Pedicle preparation was performed with the curved instrument that measures the conductivity. Each pedicle preparation was filmed using a 10 mm endoscope, with 0° angulation, linked to an arthroscopic column. We used an endoscope instead of a professional camera because it can be brought closer to the surgical site to see more details without compromising sterility conditions. Conductivity values were collected via a Bluetooth application. Recorded videos were synchronized with conductivity data via the OBS Studio application®. The trajectory was checked with a rounded head and soft feeler to rule out a breach. In the end, the screw was introduced in the pedicle. All patients were monitored with motor and sensory evoked potential. The setup is detailed in Figure 2.

The instrument is graduated with two single lines corresponding to the depth of 10 and 20 mm and a double line corresponding to 30 mm. Depth measurement was essential to match each conductivity value, at a time *t*, with the position of the instrument's tip in the vertebra. This matching was necessary for interpretation. For example, an increase in conductivity before the depth of 10 mm was not alerting since the entry point was prepared with a 10 mm bone awl, leaving a pocket filled with blood and consequently an area where the conductivity was high.

**Figure 3:**
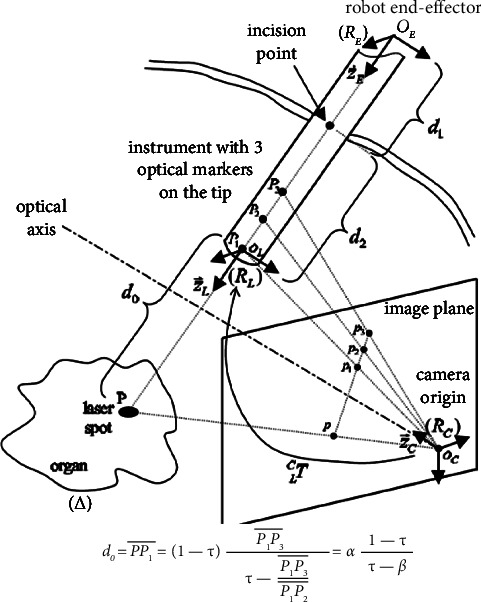
Method proposed by Krupa to calculate depth in distorted images.

Measurement of the depth was performed manually at first using the OpenCV library in the Python programming language. The position of the tip inside the vertebra was calculated by measuring the outer part since the instrument is graduated every 10 mm. Measurement was performed at a rate of one frame by second. On average, it takes 5 seconds to measure depth per frame. Consequently, if we consider a pedicle aiming duration of one minute, the time needed to measure depth every second adds up to 5 minutes. Since the camera was not perpendicular to the instrument, the resulting image is distorted. To eliminate image distortion, we used the Krupa et al.'s [18] method detailed in Figure 3.

**Figure 4:**
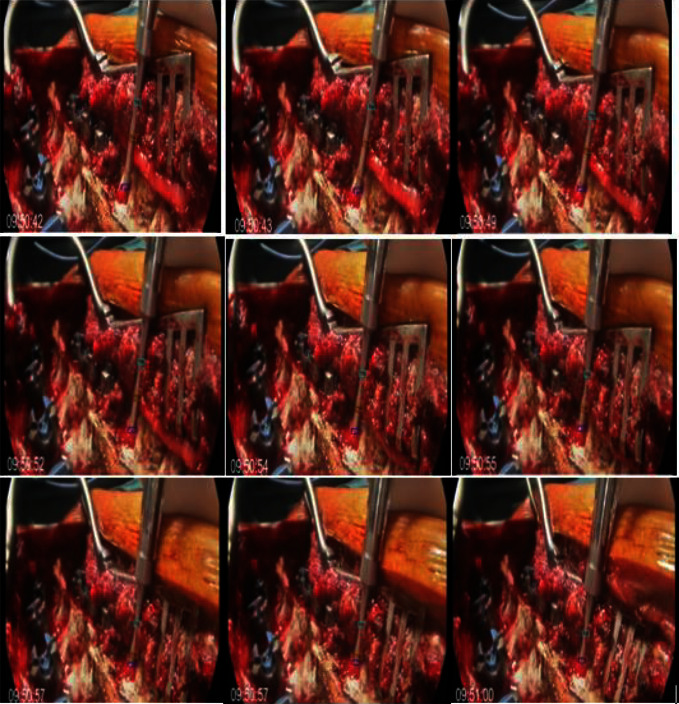
Tracking process using the CSRT method.

At each moment *t*, the depth of the instrument (*t*) was matched with its corresponding conductivity value (*s*). This was performed accurately thanks to synchronizing recorded video timestamps and the DSG application recording the signal. This technique was accurate but time consuming. So, another method was used with semiautomatic depth measurement called CSRT (Channel and Spatial Reliability Tracking). The first step was the calibration of the image. A video of a chessboard was taken from different angles. This allowed us to define the intrinsic parameters of the camera and the distance between the camera and the object in question. The code used for the calibration of the camera consisted of defining fixed points at the corners of the image, and once defined, the calibration between the real object in 3D and the image obtained will be performed. This step was essential to adjust the distortion of the picture because, as mentioned above, the camera was not always perpendicular to the plane in which the instrument was located. This technique is called semiautomatic because it requires manual positioning of the reference points. P1 and P2 were placed 1 cm apart. P3 must be set 30 mm from the instrument's tip, marked by the presence of 2 lines, not just one. P4 was placed at the point of contact of the device with the bone. A small colored rectangle will represent each point. The center of the rectangle corresponds to the chosen point. After this manual marking phase, the tracking was performed automatically. A fast displacement of the instrument (breach, instrument quickly removed for a change of direction) leads to a loss of reference points, hence the need to monitor the tracking. In this case, starting again and putting the reference points was necessary. The correlation filter improved the image quality. By way of illustration, the utilization of this semiautomatic approach for a pedicle aiming duration of 1-minute results in an average measurement time of 1.5 minutes.  Figure 4 illustrates the tracking process. The measured depths were saved in a CSV file.

To validate that the semiautomatic technique was not less accurate than the manual technique, we used the method of Bland and Altman (limits of agreement). In addition, we made a correlation between the 2 techniques and the Deming regression model.

### 2.2. Statistical Analysis

All statistical analyses were performed in the R program. The normal distribution was analyzed by the Shapiro–Wilk test. The student *t*-test (or Mann–Whitney test) and ANOVA (analysis of variance) (or Kruskal–Wallis test) were used for quantitative variables and the chi-square test for qualitative variables. A *p* value of <0.05 was considered statistically significant.

## 3. Results

Two hundred sixty-six pedicles are classified as correct and forty as breached after surgical palpation. The mean duration of pedicle preparation was 14.23 ± 2.4 seconds.

Testing of the concordance between the semiautomatic method and the manual method.

### 3.1. Correlation Coefficient

Depth in fourteen pedicles is measured with the manual technique rather than the semiautomated technique. A total of 1353 depth measurements were performed. An excellent correlation is found between the semiautomated and the manual technique, with a correlation coefficient of 0.969 (*p* value <0.001). The same results are found for each pedicle. The correlation is perfect between 10 and 20 mm. Pearson correlation coefficient detects linearity between two variables but it fails to detect disagreement in several situations [19].

### 3.2. Bland–Altman Method

**Figure 5:**
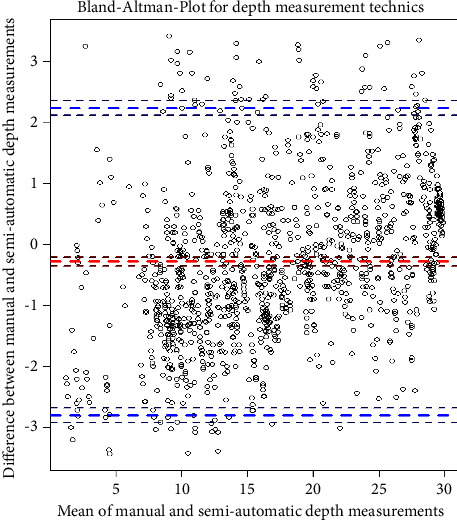
Bland–Altman method for manual versus semiautomatic methods in depth measurement.

The Bland–Altman method (limits of agreement) is the most popular method used to measure agreement [20]. This study's mean difference is −0.279 mm with a 95% confidence interval of [−0.349, −0.208]. The upper limit of agreement is 2.241 and 95% CI is [2.119, 2.362]. The lower limit of agreement is −2.799 and 95% CI is [−2.920, −2.677] (Figure 5). The distribution of the means indicates that there is no systematic bias in the semiautomatic method.

The mean difference is meager, so the manual and semiautomatic methods agreement is reasonable. The upper limit of agreement is almost 2 mm, which is clinically acceptable according to the breach classification system. The lower limit is higher. This agreement is performed for each pedicle separately.  Table 2 shows the mean difference and the upper and lower limits. When the displacement of the probe is brutal, such as in the case of a breach, the upper and lower limits are higher, so the measurement should be monitored continuously.

### 3.3. Regression Model

**Figure 6:**
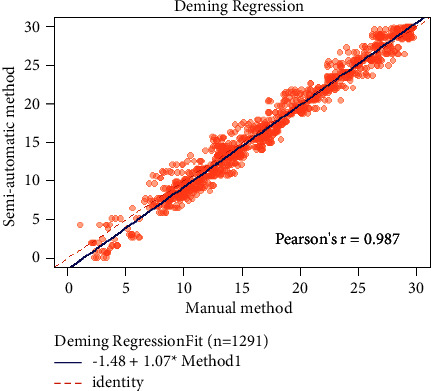
Deming regression to test agreement between manual and semiautomatic methods.

Assuming that there is uncertainty in manual method depth measurements, a Deming regression analysis is performed. The slope is 1.0686 with a standard error of 0.0047. The intercept is −1.482731 with a confidence interval of [−1.671, −1.294]. These results demonstrate a correct agreement between the manual and semiautomatic methods. The results of the Deming regression are summarized in Figure 6.

### 3.4. Analysis of Conductivity Measurements

**Figure 7:**
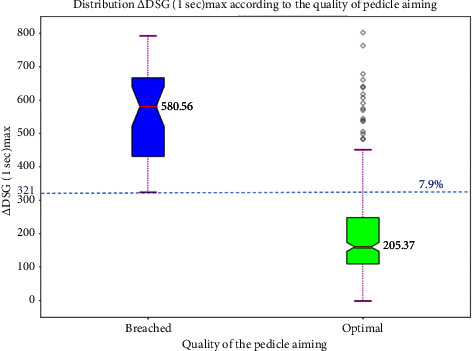
Boxplot showing the distribution of Δ (1 sec) max according to the quality of pedicle aim.

Since the electrical conductivity differs between patients, the absolute value of electrical conductivity cannot be used to differentiate spongious from liquids. For this, the conductivity gradient in one second is calculated for each pedicle (Δ_1sec_ = DSG_t_–DSG _t-1_). The means of Δ_1sec_ are 205.37 mV in the optimal group and 580.56 mV in the breached group (*p* < 0.05). To differentiate between an optimal and a breached pedicle, the maximum Δ_1sec_ (Δ_1sec_max) value is calculated for each pedicle. No breached pedicle has a Δ_1sec_max of < 321 mV and 7.9% of optimal pedicles have a Δ_1sec_max > of 321 mV (Figure 7).

## 4. Discussion

Electrical bioconductivity of tissues is already a mature technology that prevents cortical violation during pedicle drilling in the spine and in sacroiliac screws [14, 21]. These studies are based on retrospective analysis of screw placement after pilot hole drilling with subjective human interpretation of the electrical conductivity measurement device and with X-rays or CT scans without analyzing the absolute values of the conductivity signal. This study establishes a protocol to measure electrical conductivity in real time using computer vision.

Three methods are used to detect agreement between two techniques of depth measurements. Pearson's correlation coefficient is helpful to measure if two variables are linearly related. The main drawback of Pearson's estimator is that it measures the linearity between two variables, so it fails to detect disagreement in many situations, such as a change in the scale or if the samples differ by a translational term. In addition, it is affected by the variability of the measurements; the higher the variability, the higher the correlation. The Bland–Altman method (limits of agreement) is the most popular method to measure agreement in the medical literature [22]. The mean difference is −0.279 mm with upper and lower limits of agreement of 2.241 and −2.799, respectively. These values are acceptable since a cortical violation and pedicle breaching of 2 mm is benign [8]. The Bland–Altman agreement method is suitable, assuming the manual technique is a gold standard without any measurement errors. Since this assumption is not 100% sure, a regression model is the best way to test the agreement between measurement techniques. The most suitable method, in this case, is the Deming regression [23]. This method confirms the excellent agreement between both techniques.

The gradient of the conductivity signal in one second is the best estimator of breach detection. It eliminates interindividual variability of conductivity signals. Conductivity is affected by patients' temperature, blood concentration of electrolytes, hemoglobin level, preoperative bleeding, and many other factors. For each pedicle, the first 10 mm are eliminated from the study because they correspond to the prehole prepared by the bone awl; this tunnel will be filled with blood resulting in falsely high conductivity signal values. In addition, the conductivity gradient allows real-time breach detection with the possibility of developing an algorithm for an automatic stop before the cortical violation. Da Silva et al. performed this in experimental settings [24].

Thus, assuming the conductivity gradient as a reference value, we can determine whether the aim is perfect in 92.1% of cases. Several studies discuss the incidence of asymptomatic angiomas in the vertebrae, which can reach 10% [25]. Diagnosis is mainly made by MRI of the spine [26, 27]. This high incidence of hemangioma may explain a significant increase in signal in bone in 7.9%, where the conductivity gradient cannot differentiate between an optimal and breach aim. All patients included in the study had preoperative MRIs but these are intended to visualize the spinal cord and diagnose intramedullary malformation, lipoma, attached spinal cord, or Arnold–Chiari malformation. Unfortunately, these sequences do not allow the diagnosis of intraosseous lesions. Another explanation is the presence of growth plate remnants. The cartilage is more hydrated and, therefore, more conductive.

## 5. Limitation of the Study

The classification of the pedicle aims is based on qualitative measurement and the palpation by the surgeon. There is no possibility to check a cortical violation directly like in experimental settings. No scan is performed after the operation to check cortical breaches. The results obtained are promising but they need to be validated in animal experiments to eliminate measurement bias and to have a more rigorous postoperative control (CT scan or dissection).

## 6. Conclusion

In this study, we devised a research protocol aimed at the real-time assessment of electrical conductivity in direct correlation with the depth of a surgical instrument. Our semiautomated approach not only matches the precision of the manual method for instrument depth measurement but also proves to be more time efficient.

Furthermore, apart from validating the method's utility, this research underscores its potential in breach detection through the analysis of electrical conductivity values. This study represents a pioneering effort, as it is the first to utilize conductivity values instead of relying on auditory feedback as traditionally performed.

## Figures and Tables

**Table 1:** Demographic characteristics of the population.

	Mean	Sd
Age (y)	14.71	1.86
Height (cm)	157.62	10.35
Weight (kg)	50.79	13.59
Body mass index (BMI) (kg/m^2^)	20.11	3.4
Thoracic Cobb angle (°)	54.52	18.13
Lumbar Cobb angle (°)	32.81	25.47

**Table 2:** Summary of agreement limits in each pedicle.

Pedicle	Lower limit	Upper limit	Mean difference
Pedicle 1	−4.258	3.855	−0.202
Pedicle 2	−3.561	5.045	0.742
Pedicle 3	−3.496	4.039	0.272
Pedicle 4	−2.333	3.515	0.591
Pedicle 5	−4.942	1.667	−1.637
Pedicle 6	−3.294	0.904	−1.195
Pedicle 7	−2.319	2.495	0.088
Pedicle 8	−1.614	1.622	0.004
Pedicle 9	−2.199	2.751	0.276
Pedicle 10	−4.330	5.284	0.477
Pedicle 11	−3.527	2.587	−0.470
Pedicle 12	−2.339	2.379	0.020
Pedicle 13	−2.576	1.697	−0.439
Pedicle 14	−4.063	4.730	0.333

## Data Availability

The patient data used to support the findings of this study are restricted by the hospital's ethics board in order to protect patient privacy. Data are available on request from the corresponding author for researchers who meet the criteria for access to confidential data.
